# Robotic Assistance for Upper Limbs May Induce Slight Changes in Motor Modules Compared With Free Movements in Stroke Survivors: A Cluster-Based Muscle Synergy Analysis

**DOI:** 10.3389/fnhum.2018.00290

**Published:** 2018-08-15

**Authors:** Alessandro Scano, Andrea Chiavenna, Matteo Malosio, Lorenzo Molinari Tosatti, Franco Molteni

**Affiliations:** ^1^Intelligent Industrial Systems and Technologies for Advanced Manufacturing, Italian National Research Council, Milan, Italy; ^2^Rehabilitation Presidium, Valduce Ospedale Villa Beretta, Costa Masnaga, Italy

**Keywords:** muscle synergies, centroids, synergy clustering, spatial synergies, temporal components, directional tuning, stroke, robotic assistance

## Abstract

**Background:** The efficacy of robot-assisted rehabilitation as a technique for achieving motor recovery is still being debated. The effects of robotic assistance are generally measured using standard clinical assessments. Few studies have investigated the value of human-centered instrumental analysis, taking the modular organization of the human neuromotor system into account in assessing how stroke survivors interact with robotic set-ups. In this paper, muscle synergy analysis was coupled with clustering procedures to elucidate the effect of human-robot interaction on the spatial and temporal features, and directional tuning of motor modules during robot-assisted movements.

**Methods:** Twenty-two stroke survivors completed a session comprising a series of hand-to-mouth movements with and without robotic assistance. Patients were assessed instrumentally, recording kinematic, and electromyographic data to extract spatial muscle synergies and their temporal components. Patients' spatial synergies were grouped by means of a cluster analysis, matched pairwise across conditions (free and robot-assisted movement), and compared in terms of their spatial and temporal features, and directional tuning, to examine how robotic assistance altered their motor modules.

**Results:** Motor synergies were successfully extracted for all 22 patients in both conditions. Seven clusters (spatial synergies) could describe the original datasets, in both free and robot-assisted movements. Interacting with the robot slightly altered the spatial synergies' features (to a variable extent), as well as their temporal components and directional tuning.

**Conclusions:** Slight differences were identified in the characteristics of spatial synergies, temporal components and directional tuning of the motor modules of stroke survivors engaging in free and robot-assisted movements. Such effects are worth investigating in the framework of a modular description of the neuromusculoskeletal system to shed more light on human-robot interaction, and the effects of robotic assistance and rehabilitation.

## Introduction

Stroke is the leading cause of severe, long-term disability among adults (Pollock et al., 2014). After a stroke, brain damage can involve both the somatosensory and the motor areas. A recent review (Hughes et al., 2015) highlighted that up to 75% of stroke survivors suffer from proprioceptive function deficits, and about 80% from motor deficits (Pollock et al., 2014). Many approaches have been developed to deal with motor impairment after stroke, and recent guidelines for adult rehabilitation support the validity of some methods for upper extremity recovery. Examples include: functional task practice, which is “recommended”; robotic therapy, which is considered “reasonable”; and somatosensory training, which “may be considered for application” (Winstein et al., 2016). Robotic rehabilitation is one of the most promising and expanding techniques for promoting neurological recovery, as reported in Krebs and Volpe (2015). Robotic training can stimulate motor and proprioceptive responses that lay the foundations for a correct perception of space, and the self-awareness needed to purposefully execute functional movements, as reported by Colombo et al. (2016), who correlated improvements in proprioception with motor recovery.

State-of-the-art studies on the efficacy of robotic training conducted on a broad sample of patients—using clinical scales as the primary outcome (Lo et al., 2010), or instrumental clinical tests (Carpinella et al., 2014)—and comprehensive reviews on robotic rehabilitation (Mehrholz et al., 2012, 2015; Maciejasz et al., 2014) found robotic training only slightly more effective than conventional therapy. Some authors reported that upper limb functionality improved by the end of the robotic treatment (Carpinella et al., 2012), but Kwakkel et al. (2008) found comparable improvements between robotic and traditional therapy in terms of upper limb recovery. Very often, the studies considered in the reviews relied on clinical scales to assess improvements after the therapy, but these outcome measures may not be sensitive enough to capture the real differences obtained. Other methods, such as kinematics and electromyography, can be used to assess human-robot interaction in an effort to clarify the underlying motor control mechanisms.

Thus, although the use of robots for motor and proprioceptive recovery has been widely discussed in the literature, a thorough understanding of its potential is still lacking. In addition, the effects of human-robot interaction have been investigated mainly on clinical scales, while less attention has been paid to applying advanced techniques, such as electromyography (EMG) analysis. Many theories on motor control agree on the key role of the neurally-encoded representation of movement, in the form of a limited set of motor modules that account for a variety of motor tasks (Wolpert and Kawato, 1998; Wolpert et al., 1998; Feldman and Levin, 2009; Bizzi and Cheung, 2013). Since robotic training is supposed to have influence in reshaping the neural pathways underlying a movement, a detailed analysis of these motor modules may add valuable insight on human-robot interaction and the efficacy of robot-assisted rehabilitation.

A useful framework for conducting a module-based assessment is muscle synergy analysis, which is one of the state-of-the-art analytical methods for characterizing EMG activity. The core hypothesis behind muscle synergy analysis is that the central nervous system (CNS) exploits motor abundance (i.e., the redundancy of actuators with respect to the joints being actuated) to simplify the motor control problem. The synergy-based approach assumes that the CNS organizes a motor control problem by exploiting a limited number of modules, defined as muscle synergies, i.e., coordinated activation patterns of groups of muscles that share the same control signal. This approach implies that muscle redundancy is not a source of computational burden (Latash, 2012). In fact, a reduction in the number of motor commands lies at the basis of the modular organization in the neural encoding of motor synergies. Each motor module may comprise an invariant spatial synergy (a group of co-activating muscles), modulated by a time-variant component. As in other models, each module consists of a time-variant waveform that can be shifted in time, and modulated in amplitude (d'Avella et al., 2006). In the literature, many factorization algorithms have been used for synergy extraction (Tresch et al., 2016). One of the most often used is nonnegative matrix factorization (NMF; Lee and Seung, 2001). While a remarkable amount of information on muscle synergies is available in studies on animals (d'Avella et al., 2003; Overduin et al., 2015), and on healthy people (d'Avella et al., 2006, 2008), fewer studies have applied the muscle synergy framework to assessing neurological patients' motor control. Comprehensive upper-limb mappings revealed an altered recruitment pattern in mildly impaired stroke survivors, while the composition of their muscle synergies was preserved (Cheung et al., 2009). In more severely impaired patients, the spatial components of these synergies may be altered due to fractionation and merging (Cheung et al., 2012). Fractionation consists in a splitting of one apparently-healthy synergy into two or more. Merging, on the other hand, consists in the expression of two or more synergies in a single pattern, and it is typical of very low-functioning patients. Scano et al. (2017) found that a small population of stroke survivors could be correlated with a limited set of patterns that were partially related to their motor functionality. Lunardini et al. (2017) conducted a muscle synergy analysis on the upper limb muscles of children with dystonia and age-matched healthy controls while they completed various writing tasks. Their results suggest that dystonic children should have access to an intact set of synergies with a normal composition, and that their aberrant muscle activity is due to an abnormal recruitment of intact motor modules (Safavynia et al., 2011). Several efforts have been made to characterize lower limb motor modules in pathological conditions too. For instance, Clark et al. (2010) demonstrated a reduction in the number of modules underlying walking in stroke survivors. A study on patients with multiple sclerosis found that the number of muscle synergies supporting their gait was comparable with that of healthy controls, and alterations were identified more on the timing of their activation than on the composition of the synergies (Lencioni et al., 2016).

In short, the results emerging from the literature go to show that individuals with motor impairments can have a variety of muscle synergy alterations that are not easy to classify. It is nonetheless essential to gain a better understanding of these changes and the pathologies behind them in order to provide the best therapy and support.

While refined studies on robotic rehabilitation have conducted standard clinical assessments on large samples of patients (Lo et al., 2010), very few studies have coupled muscle synergies and robotic therapy to test the potential of a motor modules analysis for assessing the effects of human-robot interaction on patients with motor impairments. One such study on a limited sample of patients concluded that the treatment prompted patients to modify the coordinated activity of muscle groups, though the reorganization of the rules underlying motor control was characterized by a significant inter-patient variability (Tropea et al., 2013). Other studies discussed the use of muscle synergies and provided detailed accounts of interactions with rehabilitation devices (Coscia et al., 2014; Pirondini et al., 2016; Chiavenna et al., 2018), but their analyses only concerned healthy people.

Many experimental models involving muscle synergies have focused on trying to map the spatiotemporal features of motor modules involved in a variety of directional movements, comprehensively exploring the workspace. The purpose of the present study, on the other hand, was to extract the salient features of human-robot interaction by adopting the more restrictive constraints of our experimental design. Given the previously-mentioned premises, and the clinical approach of this study, the aim was not to comprehensively map the upper limb motor modules of stroke survivors, but to see which modules are modified by interaction with a robot, and when, in a specific gesture that might be the object of neurorehabilitation therapy (a hand-to-mouth movement, in this case). Following up the few available reports on the topic, the aim of this paper was therefore to examine the effects of human-robot interaction on stroke survivors' upper limb motor modules during robot-assisted movement, focusing on whether, and when, robotic assistance might alter the motor modules underlying these movements. The authors hypothesized that interaction with a robot could induce changes in the muscle synergies of stroke survivors. This hypothesis was tested by comparing the repertoire of motor modules detectable in two conditions—during free and robot-assisted movement—in terms of spatial synergies, temporal components, and directional tuning, as explained in detail in section Materials and Methods.

## Materials and methods

**Figure 1:**
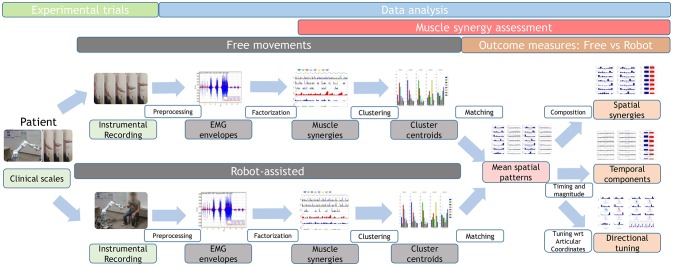
Study workflow. Twenty-two stroke survivors were recruited. They were administered the Fugl–Meyer Assessment, then they performed free and robot-assisted repetitions of the HTMM. For each of the two experimental conditions, muscle synergies were extracted using NMF. Each dataset was analyzed with the k-means clustering algorithm, and mean spatial synergies (centroids) were extracted. The two centroid datasets were matched and compared in terms of spatial composition, temporal components, and directional tuning.

A brief overview of the study is shown in Figure 1, and further details are provided below.

### Setting

The study took place at the Robotic Laboratory run by the Consiglio Nazionale delle Ricerche at the Presidio di Riabilitazione dell'Ospedale Valduce Villa Beretta, Costa Masnaga (LC), Italy.

### Participants

Twenty-two stroke survivors with chronic impairments (more than 6 months after they experienced their stroke) were recruited for the study. All patients had motor deficits in one upper limb. Eligibility criteria included a thorough understanding of the tasks to perform. The patients had different degrees of upper limb impairment, quantified with the Fugl–Meyer Assessment (FMA), Section A-D, in the range of 12/66 to 64/66. The patients' demographic and clinical characteristics are listed in Table 1, together with their FMA scores.

**Table 1:** Summary of patients' demographic data.

**Patients**	**Months since stroke**	**Impaired limb**	**Dominant hand**	**Type of stroke**	**Fugl-meyer assessment (Sections A–D)**
15M – 7F	42.1 ± 47.3	12R−10L	22R	18 ischemic 4 hemorrhagic	44.3 ± 15.7

### Equipment and motor task

**Figure 2:**
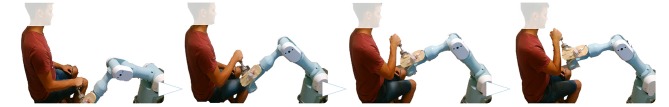
The robotic set-up, comprising a Mitsubishi Pa10 robot and a handle with a revolute joint is shown during the execution of the hand-to-mouth movement (split in four frames representing progressive phases of the movement).

The robotic set-up included a Mitsubishi Pa10-7, which is a robot with 7 degrees of freedom used to perform 3D movements. The robot was fitted with a force/torque sensor on the handle, and virtual safety walls were used to avoid unwanted contact with the user. A graphic user interface displayed the forces the user brought to bear on the handle in real time, and the position of the handle in the workspace was tracked. The handle consisted of a metal bar covered with a soft fabric and designed to be easy to grip, even for severely impaired patients. The handle was connected to the robot via a revolute joint that enabled rotation on one axis allowing for an appropriate orientation of the hand toward the mouth (Figure 2). The angle of the revolute joint developed during the movement was not monitored. In the present work, the task used to investigate muscle synergies involved a hand-to-mouth movement (HTMM), as shown in Figure 2.

In this study, the HTMM was chosen as paradigmatic of a fundamental movement needed in daily life, in gestures such as dressing or eating. The HTMM may be less burdensome than movements involving a greater contribution of force against gravity. In fact, the focus is to coordinate distal joints (the elbow and wrist) properly in order to proceed correctly toward the target (the mouth), also emphasizing the role of proprioceptive feedback in relation to the motor output. The main requirement of the task involves flexing the elbow against gravity, so spasticity or strength deficits on the elbow agonist muscles (such as the biceps or triceps) can drastically interfere with performance during its execution (Bohannon et al., 1991; Crea et al., 2017; Posteraro et al., 2017). Elbow flexion is coupled with an internal rotation and slight elevation of the shoulder. In the final part of the HTMM, a limited shoulder flexion and adduction are needed too, along with a slight forearm supination to orientate the hand correctly toward the mouth (Gopura et al., 2010).

The HTMM is therefore a functional gesture that simultaneously involves multiple-joint coordination, awareness of the body schema, and self-perception. Bearing in mind that movements toward the body are rarely assessed in the literature on motor control and learning, the authors considered the HTMM a good candidate for examining the human-robot interaction process and judging its potential for inducing neuroplasticity and motor recovery.

During the trials, patients sat on a stool with their hand resting on their thigh, and they were asked to bring their hand up to their mouth, as described in the protocol, 10 times at their own pace. The HTMM was first executed freely, then repeated with the robot. The curvilinear abscissa of the trajectory was recorded under manual guidance, then customized to fit the patients' anthropometric measures (scaled by anthropometric coefficient), and reproduced with a bell-shaped velocity profile. The trajectory could be implemented in the event of deficits of the right or left limb, and each patient trained their impaired limb. During the execution of the movements, the robot was set in active mode, i.e., the trajectory was predetermined, and no interaction with the handle could modify the law governing the motion. This was done to enable even the severely impaired participants to complete the range of motion of the gesture, and to standardize the execution of the robot-assisted movements. The use of the active mode was also supported by the findings of previous similar studies that, as far as motor modules are concerned, passive arm movements have much the same effects as fully supported ones because the same brain networks are involved during passive and active movements (Pirondini et al., 2016). Patients were nonetheless asked to participate in the movement, following the robot as if they were completing the movement independently, and minimizing the forces of interaction with the handle. This requirement meant that patients had to: (1) support the weight of their arm; (2) not push the handle; and (3) not be carried by the handle. Patients' active contribution was also required in that they had to actively guide the handle toward their mouth to complete the gesture. Since these requirements were not easy to follow, especially for the severely impaired patients, a short period (lasting about 10 min) of gradual adaptation to interaction with the robot was allowed, during which patients could practice grasping and holding the handle, and supporting the weight of their arm, to the best of their ability. This patient training phase was supported, and could be monitored, with the aid of an online visualization of the forces being exerted and a vocal feedback to patients.

During the trials, instrumental data recordings were obtained with 6-TVC and 8 s-EMG BTS Smart-D systems, for collecting kinematic and EMG data, respectively.

During both free and robot-assisted movements, kinematic data were recorded (at a sampling rate of 140 Hz) for vertebrae D5 and C7, the acromion, the elbow epicondyle, and the styloid process of the ulna. Surface EMG recordings (sampling rate = 1000 Hz) were obtained on the following muscles: upper trapezius (Tp), pectoralis major (Pt), anterior deltoid (Da), middle deltoid (Dm), posterior deltoid (Dp), triceps brachii caput medialis (Tr), biceps brachii caput longus (Bc), and brachioradialis (Br).

### Patients' clinical assessment: clinical scales

Clinical examinations were performed by a physical therapist using the FMA (Potter et al., 2011). This is a stroke-specific, performance-based scale belonging to the body function domain of the ICF model, and designed to assess motor functioning, balance, sensation, and joint functioning in patients with post-stroke hemiplegia. Only the upper extremity motor section of the FMA (scale 0-66, where 66 = no motor deficits) was used in the present study.

### Kinematic analysis

**Figure 3:**
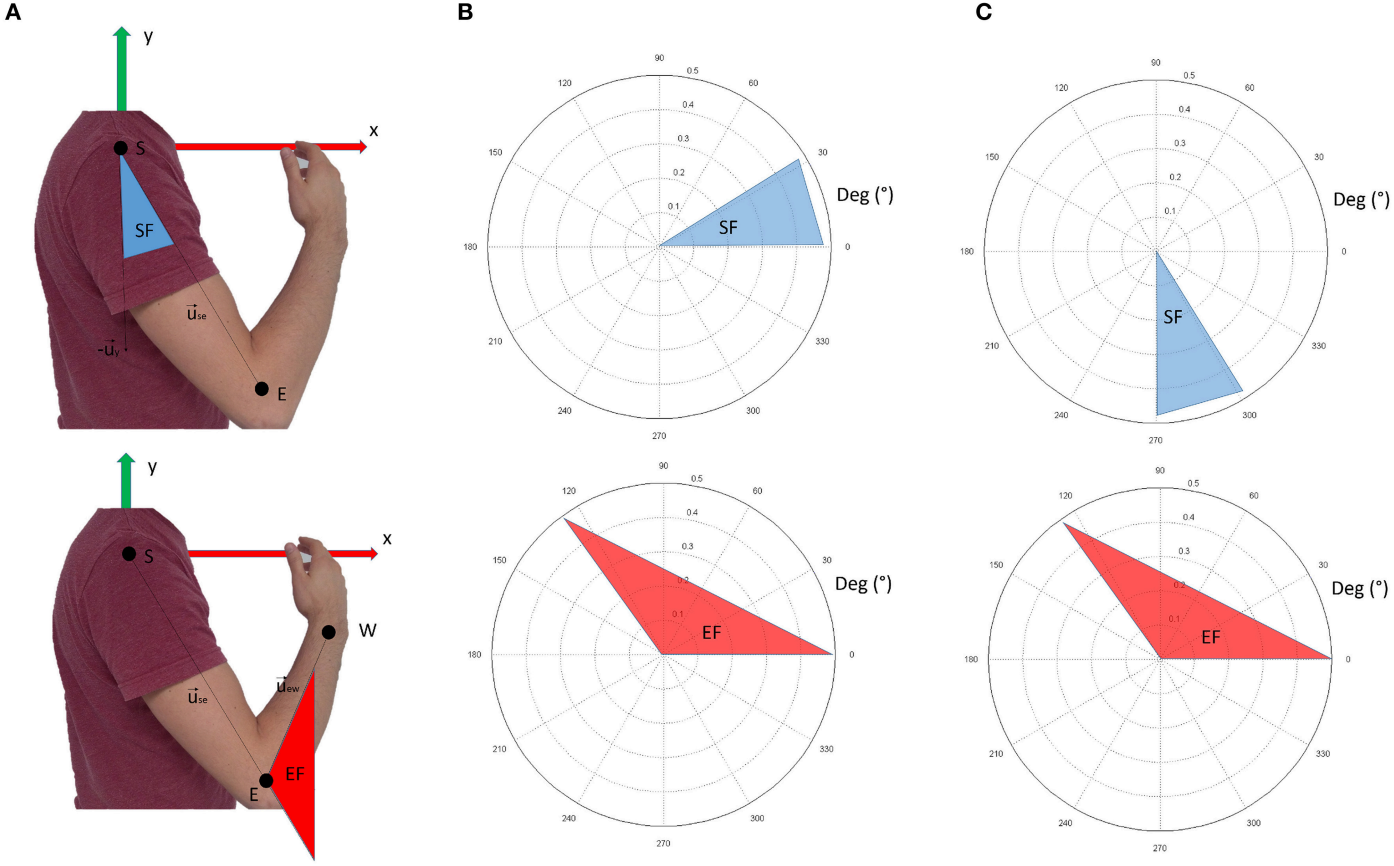
Panel **(A)** shows the nomenclature for kinematic computations. Panel **(B)** shows the conventions adopted in this study. The shoulder flexion angle is projected in the sagittal plane; 0° indicate no flexion (arm leaning along the body), while 90° indicate the shoulder flexed so that the arm is elevated frontally. The elbow angle is 0° if the arm and forearm are aligned. Positive angles indicate when the forearm is flexed. In panel **(C)**, the conventions for visualization of the results are reported. In respect to panel **(B)**, shoulder flexion angles are ofsetted (+270°). This convention was chosen to facilitate visualization of the movement when considering synergy directional tuning.

Data from retro-reflective markers were filtered with a low-pass, 3rd-order Butterworth filter with a frequency cut-off set at 6 Hz. These data were used to compute the following intrinsic body coordinates (expressed as articular angles according to the notation shown in Figure 3):

shoulder flexion angle, projected into the sagittal plane (x-y of the reference frame in Figure 3A):

(1) $$\begin{array}{r} SF=a\;cos\left(\vec{u_{SE}}\;\cdot \;\vec{u_{x}}\right) \end{array}$$

elbow flexion angle (computed in 3D coordinates):

(2) $$\begin{array}{r} EF=a\;cos\left(\vec{u_{SE}}\;\cdot \;\vec{u_{EW}}\right) \end{array}$$

where ${\vec{u}}_{\text{se}}$ is the unit vector of the shoulder-elbow anatomical axis (arm), ${\vec{u}}_{\text{x}}$ is the unit vector of the x axis of the reference framework, and ${\vec{u}}_{\text{ew}}$ is the unit vector of the elbow-wrist anatomical axis (forearm).

Figure 3B graphically represents the kinematic angles computed. In the Results section, however, the shoulder flexion angle was plotted with a +270° offset (as depicted in Figure 3C) to facilitate the graphical representation of the results (especially for directional tuning).

### Synergy extraction

Kinematic recordings were used to identify phases of the movements. Only forward phases (while the hand was approaching the mouth) were considered for our analysis, and identified with an algorithm for automatic phase detection based on the derivative of the wrist vertical coordinate. For each trial, data from the 8 sEMG channels were high-pass filtered at 50 Hz (Butterworth filter, 5th order) to remove motion artifacts, then rectified and low-pass filtered with a cut-off frequency of 10 Hz (Butterworth filter, 5th order) to remove noise and obtain EMG envelopes (Pirondini et al., 2016). Then the EMG envelopes of each HTMM were aligned and resampled (1000 samples per phase) to enable their direct comparison.

Let *nSub* = 22 be the number of patients involved, *cond* = 2 the number of experimental conditions (free and robot-assisted), *rate* = 1000 the resampling size of each aligned movement, and *nMus* = 8 the number of muscles considered. The EMG envelopes from each experimental condition were pooled together in a group of *nSub* × *cond* aggregated matrices (each *rate* × *nMus* in size), and synergies were extracted using the NMF algorithm (Lee and Seung, 2001) to break down each of the aggregated matrices. The NMF breaks down the EMG aggregated matrix into the product of two matrices, the first representing time-invariant, neural-coded synergies (*w*_*i*_), and the second representing time-variant activation commands for each synergy (*c*_*i*_), as in Equation (3):

(3) $$\begin{array}{r} EMG\left(t\right)=\sum_{i\;=\;1}^{N}\;c_{i}w_{i} \end{array}$$

where, for each of the muscles recorded, *EMG(t)* is the EMG data at the time *t*, and *N* is the total number of synergies extracted. The dimensions of the matrices *c*_*i*_ and *w*_*i*_ depend on the order of factorization *r*, which is specified by the user before synergy extraction. The matrices *c*_*i*_ and *w*_*i*_ have dimensions (*rate* × *r*) and (*r* × *nMus*), respectively. All possible orders for the factorization were tested by increasing *r* from 1 to 8 (maximum number of muscles characterizing the original dimensionality of the EMG recorded). For each *r*, the NMF algorithm was applied 1000 times to avoid local minima, and the repetition accounting for the higher variance of the signal was chosen as being representative of the order *r*. The number of synergies was chosen as the minimum *r* explaining at least 0.80 of the variance of the signal. Further synergies were only added to the dataset if increasing *r* added at least 0.05 to the overall variance explained.

### Synergy dataset clustering

Published state-of-the-art studies on muscle synergies focus on the difference between two synergy datasets (comparing movements of the more and less affected upper limbs of a stroke survivor, for instance), defining clusters and grouping spatial synergies by their composition. Each cluster is represented by a centroid, which is a mean spatial synergy comprising a group of modules that share much the same spatial composition. Cluster analysis thus enables the datasets of the extracted synergies to be reduced to a limited number of groups (or clusters), which synthetically represent the repertoire of motor modules available to the patient in each experimental condition. Then two experimental conditions are typically compared, and changes in centroid composition are used to measure the difference between the two datasets (free vs robot-assisted movements in the present study).

To obtain a synthetic representation of the motor modules representing the original datasets, clustering was applied to two aggregated matrices containing the dataset of all the extracted synergies in each of our two experimental conditions (free and robot-assisted movements). Let *n_free* be the number of motor modules extracted in free movements, and *n_robot* the number of motor modules extracted in robot-assisted movements. Clustering was applied to a “Free Movements” matrix (*n_free* × *n_Mus*), and to a “Robot–Assisted” matrix (*n_robot* × *n_Mus*). For each of the two experimental conditions, clustering was done with the k-means cluster algorithm (Matlab), which involves selecting the order of data reduction (*n_cluster*) as input. The authors opted to test solutions with *n_cluster* ranging from 2 to 20, by repeating the algorithm 1000 times and selecting, for each order, the solution with the lowest mean Euclidean distance between each centroid and the synergies it represented.

Then the choice of the appropriate number of clusters for each experimental condition was based on:

1. the most parsimonious number of clusters needed to ensure an adequate power (i.e. the lowest possible number of clusters assuring a reasonable descriptive accuracy);
2. the mean Euclidean distance of the synergies in the dataset from their centroids of order *n* had to be less than 5% of the mean Euclidean distance of the whole dataset from the centroid of order *1*;
3. single-patient cluster solutions were avoided, or limited as much as possible.

### Effects of human-robot interaction: outcome measures and statistics

To conduct a quantitative assessment of the differences between the two experimental conditions (free and robot-assisted movements), the clusters obtained were compared to identify the modifications induced by robotic assistance. Three main characteristics were considered - spatial synergy composition, temporal components, and synergy directional tuning - as described below.

Spatial synergy composition indicates which muscles are involved, and with which magnitude, in a neurally encoded coordinated module (Bizzi and Cheung, 2013). Each cluster is represented by a spatial synergy, expressed by an 8-dimensional, normalized vector, indicating the mean spatial synergy of groups of similar modules found in one of the experimental conditions. In this study, centroids (mean spatial synergies) found for each of the two experimental conditions were first matched pairwise, based on their similarity, as assessed with the dot product. Then, the dot product was used to assess the similarity of the spatial organization of each cluster pair. A dot product equal to one indicates two identical synergies, while 0 indicates orthogonality. To adopt a reference for this similarity, previous studies considered 0.90 as a threshold for a strong similarity, and 0.75 as indicative of a good similarity (Cheung et al., 2009). No further statistics or analyses were obtained on the spatial synergies.

Temporal components (or activations) are the time-variant signals used by the CNS to coordinate a group of muscles, expressed in relation to the phase of a movement (0-100%). The implicit assumption for the comparison of temporal components is that each pairwise group of temporal components (in free and robot-assisted movements) refers to the same spatial module. This assumption is acceptable when the two mean spatial modules in the pair are very similar. Two parameters were considered to judge the similarity of the mean temporal components in each cluster pair: the “shape” of the mean temporal component in relation to the phase of the movement, assessed with Pearson's correlation coefficient R (p indicates the statistical significance of the test); and the overall integral of the activation profiles (accounting for its magnitude). For each pair of centroids, differences in magnitude were tested by comparing the magnitude of each group of temporal components (free vs robot-assisted movements) with the Wilcoxon signed rank non-parametric test (alpha = 0.05).

Directional tuning entails mapping a spatial synergy with the relevant motor output variables it generates, in much the same way as proposed in d'Avella et al. (2006). This procedure can link each spatial synergy to its Cartesian directionality, as in previous works [d'Avella et al. (2006), Tropea et al. (2013), and Pirondini et al. (2016)], or be referred to intrinsic body coordinates (articular angles). Given the nature of the HTMM, mean spatial synergies and activation profiles were coupled with intrinsic kinematics, considering the shoulder flexion angle and the elbow flexion angle. The mean directional tuning associated with each centroid was computed by considering the articular angles corresponding to the barycenter of the weighted mean temporal component relative to that centroid. Two directional tunings were thus computed for each of the centroids found in each of the two experimental conditions: one for shoulder flexion and one for elbow flexion. Then, for each pair of centroids, the difference in directional tuning for each of the two angles was tested with the Wilcoxon signed rank non-parametric test (alpha = 0.05).

## Results

All patients were able to complete the trial, each according to their motor functionality. The results presented in sections Spatial synergies, Temporal components, and Synergy directional tuning are also listed in Table 2.

**Table 2:** Summary of results.

**Components**		**Spatial synergies**	**Temporal components**			**Directional tuning**			
		**Dot Prodict(Similarity)**	**Shape: Pearson's correlation**	**Magnitude: integral**	**Magnitude: statistics**	**Shoulder flexion (deg)**	**Statistics**	**Elbow flexion (deg)**	**Statistics**
Cluster ID Free	1	0.972	*R* = −0.527 *p* < 0.001	0.0475	*h* = 0 *p* = 0.79	29.93	*h* = 0 *p* = 0.11	95.38	*h* = 0 *p* = 0.79
Cluster ID Robot	1			0.0559		23.59		91.16	
Cluster ID Free	2	0.973	*R* = 0.900 *p* < 0.001	0.1151	*h* = 0 *p* = 0.08	24.97	*h* = 0 *p* = 0.15	102.85	*h* = 0 *p* = 0.97
Cluster ID Robot	2			0.0739		28.61		99.25	
Cluster ID Free	3	0.959	*R* = 0.761 *p* < 0.001	0.1560	*h* = 0 *p* = 0.23	36.43	*h* = 0 *p* = 0.25	98.45	*h* = 0 *p* = 0.82
Cluster ID Robot	3			0.0920		27.75		93.22	
Cluster ID Free	4	0.982	*R* = 0.820 *p* < 0.001	0.0879	*h* = 0 *p* = 0.44	31.09	*h* = 0 *p* = 0.62	95.53	*h* = 0 *p* = 0.94
Cluster ID Robot	4			0.0630		27.02		95.65	
Cluster ID Free	5	0.959	*R* = 0.037 *p* < 0.001	0.0628	*h* = 0 *p* = 0.85	10.04	*h* = 0 *p* = 0.22	101.75	*h* = 0 *p* = 0.22
Cluster ID Robot	5			0.0471		20.81		85.61	
Cluster ID Free	6	0.898	*R* = 0.600 *p* < 0.001	0.0381	*h* = 0 *p* = 0.36	26.62	*h* = 0 *p* = 0.69	98.41	*h* = 0 *p* = 0.63
Cluster ID Robot	6			0.0546		27.63		95.05	
Cluster ID Free	7*	0.628	n.a.	n.a.	n.a.	n.a.	n.a.	n.a.	n.a.
Cluster ID Robot	7*			n.a.		n.a.		n.a.	

*The table is divided vertically into three sections. Each section shows the results of the comparisons between free and robot-assisted movements in the three global outcome domains: spatial synergies, temporal components, and directional tuning. The spatial synergies column describes the similarity of the pairwise-matched motor modules. The temporal components section is divided into three sub-sections. The shape of the mean temporal components was assessed with Pearson's correlations, reported with the p-value of the test. The second sub-section shows the integral value of the mean temporal components. The third sub-section shows the results of the Wilcoxon test on the difference in magnitude between the two experimental conditions, within each cluster. The directional tuning section shows the mean directional tuning for shoulder flexion and elbow extension, and the results of the Wilcoxon test on the difference in directional tuning between the two experimental conditions, within each cluster*.

* *The statistics for the temporal components and directional tuning were omitted because the centroids were dissimilar*.

### Synergies

**Figure 4:**
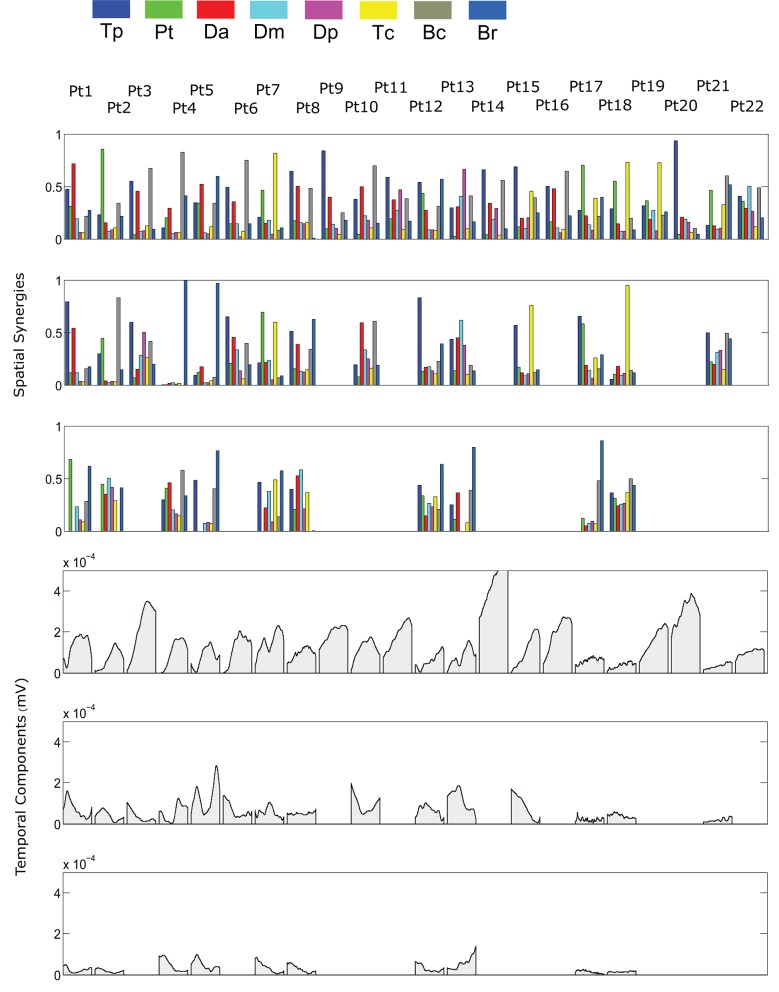
Patients' muscle synergies and their temporal components in free HTMM. Rows 1–3 show the composition of the synergies; rows 4–6 show the corresponding temporal components.

**Figure 5:**
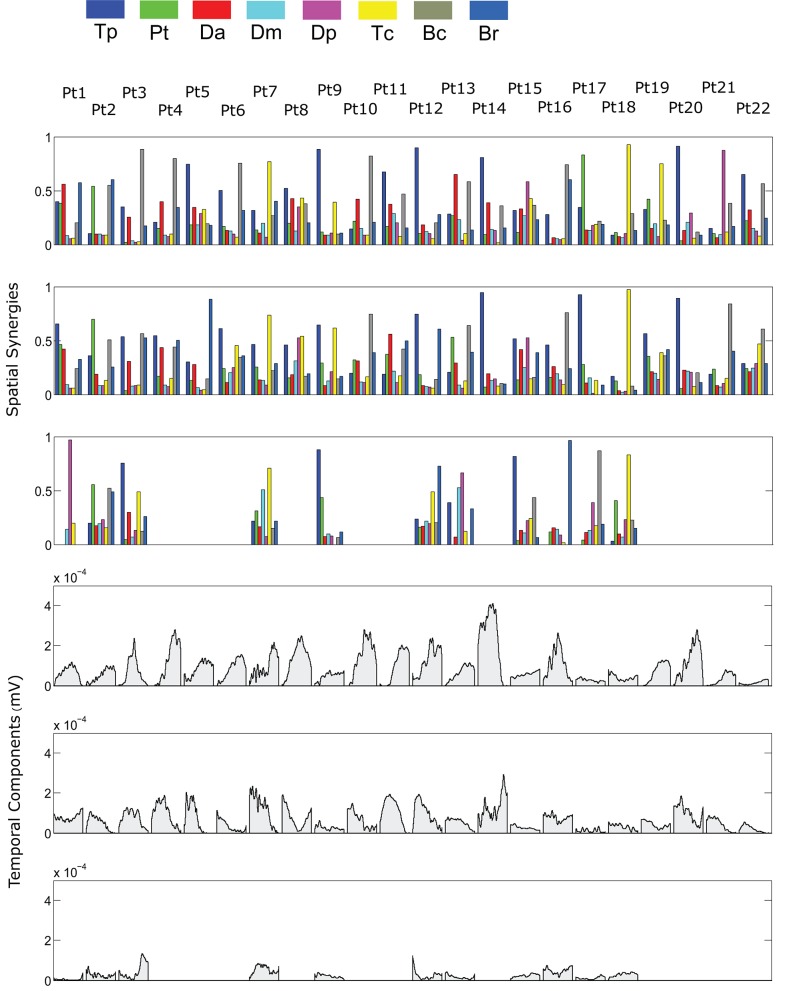
Patients' muscle synergies and temporal components in robot-assisted HTMM. Rows 1–3 show the composition of the synergies; rows 4–6 show the corresponding temporal components.

The spatial synergies extracted from the data and the temporal components, for all patients, are shown in Figure 4 (for free movements) and Figure 5 (for robot-assisted movements). Depending on the metrics chosen for synergy extraction, each patient's original EMG was reconstructed with a minimum of 1 and a maximum of 3 synergies.

### k-means clustering

**Figure 6:**
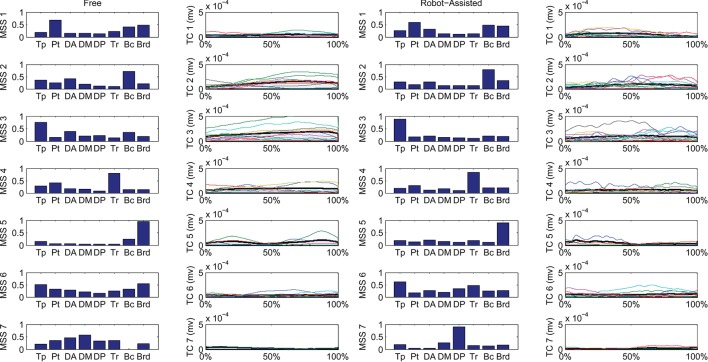
Mean spatial synergies (MSS), matched pairwise by similarity, assessed with the dot product on muscle coefficients, for both free and robot-assisted conditions, with the associated temporal components (TC).

Figure 6 shows details of the clustering procedure. Seven clusters were identified in each condition, 7 being the lowest clustering order achieving a mean Euclidean distance of less than 5% in relation to the clustering of order 1. Clusters were matched pairwise, and plotted to highlight spatial synergies and temporal components, for the free and robot-assisted HTMM, respectively.

### Spatial synergies

Table 2 shows details of the similarities between the pairs of clusters, assessed with the dot product of the spatial synergy compositions. Six out of 7 clusters were very strongly similar (dot > = 0.90): cluster 1 pairwise dot 0.97; cluster 2 pairwise dot 0.97; cluster 3 pairwise dot 0.96; cluster 4 pairwise dot 0.98; cluster 5 pairwise dot 0.96; cluster 6 pairwise dot 0.90, while the pairwise dot on one centroid was low (0.63).

### Temporal components

**Figure 7:**
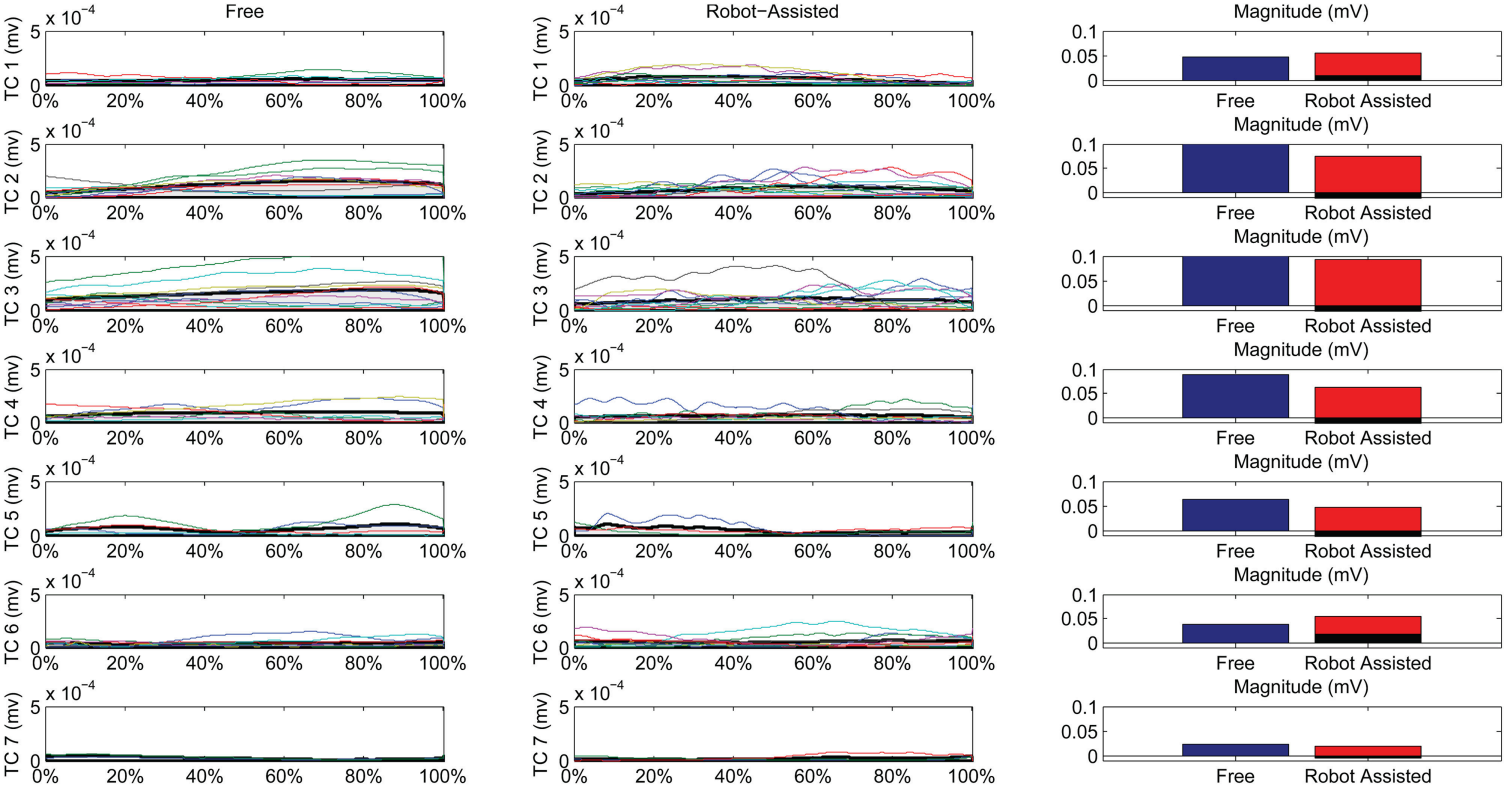
Temporal Components (TC) of muscle synergies, matched pairwise by similarity, for both free and robot-assisted conditions. The mean temporal component is highlighted (light gray plot). Bar graphs show the magnitude (integral) of the mean temporal components, comparing free and robot-assisted conditions. The black histogram highlights the difference between the magnitude of the temporal components in free and robot-assisted conditions.

Figure 7 shows details of the similarities between the pairs of clusters, assessed on the temporal components. The magnitude of the EMG associated with each spatial synergy was measured from the mean integral magnitude of each temporal component. The results indicate, for all the spatial synergies, that the magnitude of the temporal components did not change significantly when movements were robot-assisted (*p* > 0.05). Since the similarity of cluster 7 was weak, the comparisons based on temporal components were not consistent, and are consequently not reported here. For 4 of the other 6 centroids, there was a trend (though it lacked statistical significance) toward a reduction in the magnitude of the temporal components during interaction with the robot: cluster 2: 0.1151 mV (free) vs. 0.0739 (robot); cluster 3: 0.1560 mV (free) vs. 0.0920 (robot); cluster 4: 0.0879 mV (free) vs. 0.0630 (robot); cluster 5: 0.0628 mV (free) vs. 0.0471 (robot). Opposite results emerged for the other two: cluster 1: 0.0475 mV (free) vs. 0.0559 mV (robot); and cluster 6: 0.0381 mV (free) vs. 0.0546 (robot).

Table 2 also shows Pearson's correlation coefficients for each pairwise group of temporal components, accounting for the “shape” of the activations. While some of the temporal component waveforms showed positive or very positive correlations (cluster 2 = 0.90; cluster 3 = 0.76; cluster 4 = 0.82; cluster 26 = 0.60;), others showed moderately negative correlations (cluster 1: −0.577), or no correlation at all (cluster 5: 0.04).

### Synergy directional tuning

**Figure 8:**
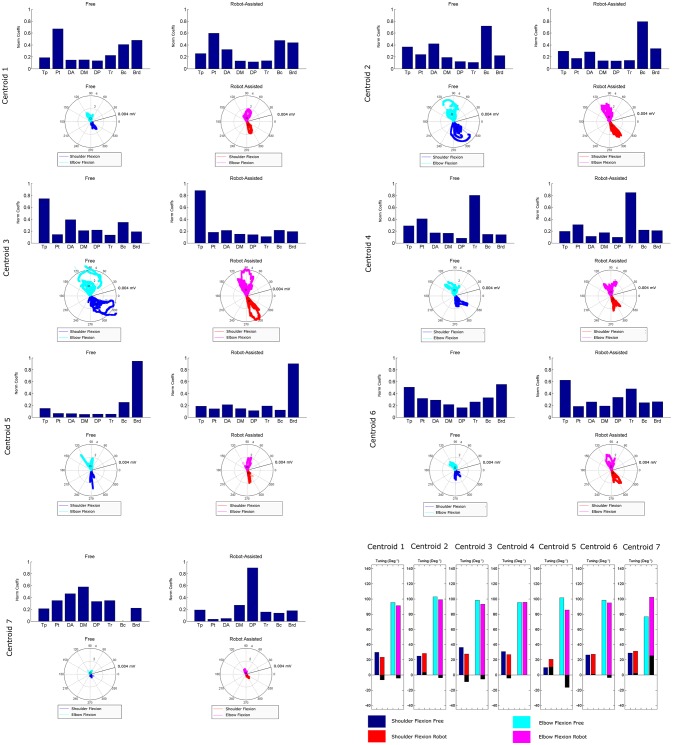
Mean directional tuning of muscle synergies for each pair of centroids. Each radar plot shows a scatter plot of the activations of each synergy centroid within the workspace, plotted versus shoulder elevation and elbow flexion, respectively, according to the convention illustrated in Figure 2C. When shoulder flexion is indicated, the radar plot should be interpreted as the projection on the sagittal plane, with movements executed by pointing rightwards. When elbow flexion is indicated, its full extension is at 0°. Blue plots indicate shoulder flexion in free movement trials; cyan plots indicate elbow flexion in free movements; red plots indicate shoulder flexion in robot-assisted trials; magenta plots indicate elbow flexion in robot-assisted tracks; squares are plotted to indicate the barycenter of the distribution. The final bar plot summarizes the mean spatial tuning for each of the intrinsic body coordinates considered (shoulder flexion and elbow flexion) and each experimental condition.

Figure 8 is a graphical representation showing details of the synergies' directional tuning. The barycenter of the temporal component activations never differed significantly (*p* > 0.05), but some modules were averagely elicited at slightly different intrinsic coordinates (articular angles). In fact, some modules were used at lower articular angles (i.e., in early phases of the HTMM): the barycenter of cluster 1 was at 29.93° shoulder flexion (SF) and 95.38° elbow flexion (EF) in free movement, and at 23.59° SF and 91.16° EF, respectively, in robot-assisted movement; the barycenter of cluster 3 was at 36.43° SF and 98.45° EF in the former case, and at 27.75° SF and 93.22° EF in the latter. On the other hand, cluster 2 (24.97° SF and 102.85° EF in free movement, 28.61° SF and 99.25° EF in robot-assisted movement) and cluster 5 (10.04° SF and 101.75° EF in the former case, as opposed to 20.81° SF and 85.61° EF in the latter) were averagely recruited at higher shoulder angles but lower elbow angles. Lastly, cluster 4 was recruited at slightly lower shoulder angles (31.09° SF and 95.53° EF in free movement, 27.02° SF and 95.65° EF in robot-assisted movement), and cluster 6 was recruited at lower elbow angles (26.62° SF and 98.41° EF in free movement, 27.63° SF and 95.05° EF in robot-assisted movement). Table 2 lists the mean directional tunings for each spatial component, matched with the shoulder flexion and elbow flexion angles.

## Discussion

### Clusters

First of all, a description of the clusters extracted is given below to highlight the function involving each of the motor modules. The clusters are described referring to Figure 6, which shows their centroids relating to free and robot-assisted HTMM.

#### Cluster 1—flexor synergy

This cluster represents the flexor synergy, and includes a relevant contribution from the pectoralis, biceps, and brachioradialis muscles. This synergy is the basic pattern underlying HTMM. It involves a slight flexion and intra-rotation of the shoulder, and flexion of the elbow and hand to reach the mouth.

#### Cluster 2—elbow flexion and gravity support at end of movement

The pattern in this cluster includes a major contribution from the biceps, supported by shoulder elevators such as the trapezius and anterior deltoid muscles. This synergy is elicited mainly at the end of the HTMM to maintain posture against gravity at both shoulder and elbow levels, and to complete the flexion of the elbow.

#### Cluster 3—proximal stabilization

This cluster includes a major contribution from the trapezius, partially supported by elbow flexor muscles. This synergy is elicited in various phases of the movement, and supports its stabilization.

#### Cluster 4—elbow co-contraction

This cluster includes a major contribution from the triceps muscle, probably supporting a co-contractile action to stabilize the forearm in various phases of the movement.

#### Cluster 5—early elbow flexion

This cluster is strongly represented by brachioradialis activity, especially at the beginning of the movement. It is probably connected to the recruitment of the forearm and hand to flex the elbow and properly orient the end effector before starting to approach the mouth.

#### Cluster 6—completion of co-contractile and stabilizing synergy

This cluster includes contributions from many muscles, which co-contract and stabilize the action, especially at the end of the movement.

#### Cluster 7—shoulder contribution

This cluster comprises two different patterns. In free HTMM, it takes effect at the beginning of the movement to start elevating the shoulder. In robot-assisted HTMM, its main contribution comes from the posterior deltoid, which slows and stabilizes the limb at the end of the movement.

### Spatial synergies

Referring to Figure 6, seven clusters could account for the original dataset of synergies extracted for the free and robot-assisted HTMM. In the present study conditions, the dimensionality of the control space was the same for the movements with and without robotic assistance. Six of the seven pairwise-matched clusters were also very similar when free and robot-assisted movements were compared (similarity > = 0.90). The only cluster pair that was different revealed an alteration of the coordinated motor modules. While the number of centroids and the sample of patients considered are too small to obtain strong statistics, it could be argued that the robot introduced a slight change in the spatial components of the motor modules compared to patients' free movements. This effect was mainly apparent in the emergence of a module featuring a marked posterior deltoid activity, toward the end of the HTMM, probably to slow and stabilize the limb (whereas the pattern in free movements involved the anterior and middle deltoid acting to elevate the limb at the beginning of the HTMM). Overall, the spatial components were altered, but only marginally. These results are consistent with other reports in the literature. Limited numbers of motor modules characterizing upper limb control were found in experimental studies comparing free vs. device-assisted movements in rehabilitation settings (Coscia et al., 2014; Pirondini et al., 2016), in patients vs. healthy controls (Cheung et al., 2009; Roh et al., 2015), and in more vs. less affected limbs (Cheung et al., 2009; García-Cossio et al., 2014). Our findings also seem to suggest that robotic assistance introduces only slight changes in the spatial synergies of stroke survivors, as seen in healthy people. In many of the above-mentioned studies on free vs. assisted movements, the dimensionality of the control was comparable in the two conditions, although Cheung et al. (2009) found evidence of motor module merging and fractionation for the more affected of two limbs in the same patient, in respect to healthy controls. In our present work, interaction with the robot did not consistently alter the patients' original spatial patterns. This result could support the choice of a robot-assisted rehabilitation program, especially when a target task has been identified and has to be repeated numerous times as part of an intensive training. In fact, the rehabilitation literature emphasizes that, while a great variety of movements should be considered to generalize the effect of training (Schmidt, 1975), mastery of a particular skill is increased if that skill is trained specifically. On the other hand, if interaction with an external device can modify muscle activation patterns, then non-therapeutic outcomes may also come to light due to the triggering of unwanted modules. In our patients, the spatial synergies proved robust across both experimental conditions, however, and this finding supports the use of robots in rehabilitation training protocols designed to improve motor functionality in specific gestures. Abilities trained by means of robotic protocols might also be transferred to free movements because the modules recruited are much the same. Though this issue needs to be tested in a complete rehabilitation protocol, previously-published findings may provide theoretical support for our results.

### Temporal components

Referring to Figure 7, we can see noteworthy differences in the correlations on the mean temporal components, aligned on the phases of the movement. Only 4/7 pairs of centroids featured positively correlated mean temporal components, while 1 showed no correlation at all, and 2 even showed negative correlations along the phases of the movement. While the lack of correlation for cluster 7 was expected (because its composition differed between the two conditions), a negative correlation also emerged in the recruiting of flexor synergy, which is an important pattern in the performance of a HTMM. Furthermore, the flexor synergy was recruited slightly earlier during the robot-assisted movement, and decreased toward the end, replaced by other patterns that refined the approach of the hand to the mouth. In patients' free movements, this synergy was elicited in a later phase, when the available range of motion had nearly been reached. The robot probably helped patients to complete the main pattern relating to the execution of the task in its earlier stages by providing support for the limb. If this finding is confirmed in a larger sample of patients, it would suggest that robots can have therapeutic effects on patients (or promote correct proprioception at least). The overall magnitude of the modules was slightly modified, even if the difference was not statistically significant. In fact, 5/7 modules were elicited to a lesser extent during interaction with the robot, indicating that the set-up was probably helping patients a little, enabling a reduction in their EMG activity. Only two patterns did not obey this rule. One was the flexor synergy, which increased in interaction with the robot (probably because the robot facilitated the start of the movement by providing support). The other concerned a co-contractile activation occurring at the end of the movement to stabilize the motor pattern. As underscored when the outcomes were discussed in terms of spatial synergies, our findings on the temporal components partially reflect the results of previous studies. Although the present study did not entail a full rehabilitation trial, the results are comparable with the report from Tropea et al. (2013), who suggested that robot therapy may induce partial modifications of the temporal components (largely depending on the motor capability of each patient). Such modifications, involving a slight (statistically insignificant) reduction in the magnitude of the modules, may be due to the support provided by the robot. Similar results were found in healthy individuals by Coscia et al. (2014), when the temporal components were scaled proportionally to the level of support provided by the device. Pellegrino et al. (2018), on the other hand, found changes in shoulder muscle activations, especially in movements toward the body. If such results are confirmed in further studies, we might conclude that interaction with the robot does not modify the modules underlying a movement, but may affect how the modules are exploited, especially in terms of timing and magnitude.

### Directional tuning

Our findings concerning mean directional tuning correlated partially with the results of our analysis on the temporal components. The two assessments differ in that, while the temporal components relate to movement phases, directional tuning captures correlations with intrinsic body coordinates or Cartesian coordinates (i.e., articular angles, as proposed in d'Avella et al., 2006). The directional tuning analysis presented in Figure 8 thus takes into account the fact that some patients did not complete the gesture. Several differences emerged in the mean tuning parameter, though they were less remarkable than those affecting the temporal components, and they never reached statistical significance. The most important difference regards the flexor synergy, which was elicited at a slightly later stage in free movements than with robotic assistance. A similar result was found on centroid 3, which was elicited for lower articular coordinates (earlier in the movement), indicating that robotic support slightly anticipated the recruiting of this module. Cluster 7 is not directly comparable between the two experimental conditions due to the different mean spatial synergies involved, but in robot-assisted movements it was recruited at a considerably later stage in the movement. Lesser effects are observable on the synergies in clusters 2, 4, and 6. Finally, cluster 5 was anticipated in the recruitment tuning associated with robot-assisted movements, corresponding to less elbow flexion than in free movements, and slightly more shoulder flexion. This anticipation is consistent with the expected role of the brachioradialis as an elbow flexor at the start of the movement. Few published studies analyzed the directional tuning of muscle synergies, some of which identified marked changes in the temporal components. Applying an 8-target training paradigm to healthy people and neurological patients before and after a course of robotic rehabilitation, Tropea et al. (2013) found that the spatial synergies were elicited with different Cartesian tunings (showing greater differences than in the present study). Such changes may be attributed to the effects of the therapy (not shown in this study) or to the different strategies used to control the robot.

### Impact on future work

The module-based approach used here to assess the effects of human-robot interaction produced some interesting insight on the control of movements. Standard approaches, such as clinical scales, provide a semi-quantitative picture of patients' status, whereas the muscle synergy approach enabled us to investigate how motor modules were organized in their spatial and temporal components. Motor module analysis offers a perspective on neuromuscular diseases that differs from those of clinical scales and kinematics because it investigates a higher hierarchical level of motor production, shedding light on neuromuscular coordination rather than on output motor variables alone. Since the aim of robotic rehabilitation is to improve motor functioning by inducing a reshaping of the neural pathways underlying a movement, muscle synergy should be seen as a primary outcome for assessing the effects of neuromotor robotic training.

Robotic training programs for the upper limbs have concentrated so far on functional tasks, or on a variety of Cartesian directions of motion (Carpinella et al., 2012). Such paradigms are now well established and have proved effective on patients, but they usually employ muscle synergies as assessment tools rather than as design elements. The potential of muscle synergies might be used instead to identify patient-specific patterns to retrieve and train with the robot's support. Different modes of assistance could be fine-adjusted to train specific synergies, or promote the use of meaningful modules. Better still, judging from the limited findings available in the literature, the timing and magnitude of the available spatial synergies might be modified to make the best of a given patient's potential. In the light of the results of this and previous studies, training might presumably also be improved by promoting muscle-synergy-based protocols coupled with other control paradigms. To give an example, patients could be exposed to different mechanical environments, as in Pellegrino et al. (2018), who reported differences in the spatial and temporal patterns of patients with multiple sclerosis compared with healthy controls. Extending this concept might lead to the generation of customized force fields to elicit or train specific motor synergies.

To conclude, the present study partially confirms previous reports in the literature, suggesting that interaction with robotic devices may induce slight alterations in the spatial motor modules of stroke survivors, as seen in healthy people. These modules may be elicited differently, however, and the timing of their activation or their directional tuning may change to some degree. All these points may point to opportunities for testing rehabilitation paradigms based on the coordination of motor synergies.

### Limitations

The present study has some limitations. The first concerns the small sample of patients tested. A larger sample would be needed to give more solidity to the cluster centroids identified. Our results therefore cannot be generalized to a variety of gestures or to the whole repertoire of synergies available to the sample of stroke survivors enrolled in this study. Second, our sample also included patients with very different motor impairments, partially weakening the strength of our conclusions. Considering the very limited knowledge available on the topic, and especially on robotic set-ups applied to patients, and bearing in mind that many previous studies suffered from the same limitations relating to different motor impairments and small sample sizes, the present findings seem worthy of interest. Third, the comparisons proposed here were limited to a single session of acquisitions, and only 8 muscles were monitored. That said, the aim of the present study was to test the potential of the muscle synergy approach for assessing patients' EMG patterns during robotic training. Our preliminary results suggest that the method could be used to examine patients interacting with robots equipped with other control schemes too, based on assist-as-needed or admittance paradigms, for instance, or devices based on not-actuated gravity support. Of course, further refinements of the analytical method may be needed before muscle synergy analysis can be applied to such paradigms because motor tasks may suffer from “fractionation” of the laws of motion (due to the assistance provided), making synergy extraction and matching more difficult, especially when severely impaired patients are involved. Such issues will be investigated in further studies.

## Conclusion

This paper addresses human-robot interaction in stroke survivors, discussed in the framework of muscle synergies. The synergies extracted were clustered and matched to enable the assessment of their spatial and temporal components, and their directional tuning in relation to intrinsic body coordinates. The analysis identified 7 clusters reflecting a sample of 22 patients. Each cluster was characterized to associate a physiological role with the centroid identified. The main finding of this work is that, in our set-up, interaction with the robot very slightly altered the muscle synergies' centroids (spatial components), temporal components, and directional tuning. Synergy analysis brought out limited, but observable differences between the two experimental conditions conditioned (free and robot-assisted HTMM). This effect is noteworthy because it confirms the feasibility of inducing neuroplasticity in stroke survivors with chronic motor impairments by designing and fine-tuning rehabilitative protocols devised to train muscle synergies. Future studies will further investigate this issue.

## Ethics statement

This study was carried out in accordance with the recommendations of local Ethics Committee at Lecco Manzoni Hospital. The protocol was approved by the Ethics Committee at Lecco Manzoni Hospital. All subjects gave written informed consent in accordance with the Declaration of Helsinki.

## Consent for publication

Written informed consent was obtained from patients for the publication of this study and any accompanying images. A copy of the written consent form is available for review by the Editor-in-Chief of this journal.

## Availability of data and material

The datasets analyzed in the present study are not publicly available, but are available from the corresponding author on reasonable request.

## Author contributions

AS designed the experiment, wrote the software for synergies extraction and clustering, performed the experimental campaign, elaborated the data, and wrote the paper. AC performed the experimental campaign, elaborated the data, and wrote the paper. MM was responsible for the Research Project that funded the work. He revised the paper and participated to data analysis. LM was the head of the CNR research group. He revised the paper and participated to data analysis. FM is the head of the Villa Beretta Hospital and participated to the conception of the evaluation system, and gave clinical interpretation to the data. All authors read and revised the manuscript critically for important intellectual content, and approved the final manuscript for publication. All authors agree to be accountable for all aspects of the work.

### Conflict of interest statement

The authors declare that the research was conducted in the absence of any commercial or financial relationships that could be construed as a potential conflict of interest.

## Abbreviations

ICF: International Classification of Functioning
CNS: central nervous system
HTMM: hand-to-mouth movement
EMG: electromyography
A: assisted
NA: non-assisted
Tp: trapezius
Pt: pectoralis
Da: anterior deltoid
Dm: middle deltoid
Dp: posterior deltoid
Tc: triceps
Bc: biceps
Br: brachioradialis
FMA: Fugl-Meyer Assessment
ROM: range of motion
Pt: patient
NMF: non-negative matrix factorization
VAF: variance accounted for
3D: three-dimensional.

## References

[B1] Bizzi E. Cheung V. C. (2013). The neural origin of muscle synergies. Front. Comput. Neurosci. 7:51. 10.3389/fncom.2013.00051 23641212 PMC3638124

[B2] Bohannon R. W. Warren M. E. Cogman K. A. (1991). Motor variables correlated with the hand-to-mouth maneuver in stroke patients. Arch. Phys. Med. Rehabil. 72, 682–684. 1859265

[B3] Carpinella I. Cattaneo D. Bertoni R. Ferrarin M. (2012). Robot training of upper limb in multiple sclerosis: comparing protocols with or without manipulative task components. IEEE Trans. Neural Syst. Rehabil. Eng. 20, 351–360. 10.1109/TNSRE.2012.2187462 22623407

[B4] Carpinella I. Cattaneo D. Ferrarin M. (2014). Quantitative assessment of upper limb motor function in Multiple Sclerosis using an instrumented Action Research Arm Test. J. Neuroeng. Rehabil. 11:67. 10.1186/1743-0003-11-67 24745972 PMC3998062

[B6] Cheung V. C. Turolla A. Agostini M. Silvoni S. Bennis C. Kasi P. (2012). Muscle synergy patterns as physiological markers of motor cortical damage. Proc. Natl. Acad. Sci. U.S.A. 109, 14652–14656. 10.1073/pnas.1212056109 22908288 PMC3437897

[B5] Cheung V. C. K. Piron L. Agostini M. Silvoni S. Turolla A. Bizzi E. (2009). Stability of muscle synergies for voluntary actions after cortical stroke in humans. Proc. Natl. Acad. Sci. U.S.A. 106, 19563–19568. 10.1073/pnas.0910114106 19880747 PMC2780765

[B7] Chiavenna A. Scano A. Malosio M. Tosatti L. M. Molteni F. (2018). Assessing user transparency with muscle synergies during exoskeleton-assisted movements: a pilot study on the LIGHTarm device for neurorehabilitation. Appl. Bionics Biomech. 2018:7647562. 10.1155/2018/7647562 29967656 PMC6008767

[B8] Clark D. J. Ting L. H. Zajac F. E. Neptune R. R. Kautz S. A. (2010). Merging of healthy motor modules predicts reduced locomotor performance and muscle coordination complexity post-stroke. J. Neurophysiol. 103, 844–857. 10.1152/jn.00825.2009 20007501 PMC2822696

[B9] Colombo R. Sterpi I. Mazzone A. Delconte C. Pisano F. (2016). Improving proprioceptive deficits after stroke through robot-assisted training of the upper limb: a pilot case report study. Neurocase 22, 191–200. 10.1080/13554794.2015.1109667 26565132

[B10] Coscia M. Cheung V. Tropea P. Koenig A. Monaco V. Bennis C.. (2014). The effect of arm weight support on upper limb muscle synergies during reaching movements. J. Neuroeng. Rehabil. 11, 1–15. 10.1186/1743-0003-11-22 24594139 PMC3996016

[B11] Crea S. Cempini M. Mazzoleni S. Carrozza M. C. Posteraro F. Vitiello N. (2017). Phase-II clinical validation of a powered exoskeleton for the treatment of elbow spasticity. Front. Neurosci. 11:261. 10.3389/fnins.2017.00261 28553200 PMC5427118

[B12] d'Avella A. Fernandez L. Portone A. Lacquaniti F. (2008). Modulation of phasic and tonic muscle synergies with reaching direction and speed. J. Neurophysiol. 100, 1433–1454. 10.1152/jn.01377.2007 18596190

[B13] d'Avella A. Portone A. Fernandez L. Lacquaniti F. (2006). Control of fast-reaching movements by muscle synergy combinations. J. Neurosci. 26, 7791–7810. 10.1523/JNEUROSCI.0830-06.2006 16870725 PMC6674215

[B14] d'Avella A. Saltiel P. Bizzi E. (2003). Combinations of muscle synergies in the construction of a natural motor behavior. Nat. Neurosci. 6, 300–308. 10.1038/nn1010 12563264

[B15] Feldman A. G. Levin M. F. (2009). The equilibrium-point hypothesis - past, present and future, in Progress in Motor Control. Advances in Experimental Medicine and Biology, Vol. 629, ed Sternad D. (Boston, MA: Springer). 10.1007/978-0-387-77064-2_38 19227529

[B16] García-Cossio E. Broetz D. Birbaumer N. Ramos-Murguialday A. (2014). Cortex integrity relevance in muscle synergies in severe chronic stroke. Front. Hum. Neurosci. 8:744. 10.3389/fnhum.2014.00744 25294998 PMC4172028

[B17] Gopura R. A. R. C. Kiguchi K. Horikawa E. (2010). A study on human upper-limb muscles activities during daily upper-limb motions. Int. J. Bioelectromagnet. 12, 54–61.

[B18] Hughes C. M. L. Tommasino P. Budhota A. Campolo D. (2015). Upper extremity proprioception in healthy aging and stroke populations, and the effects of therapist-and robot-based rehabilitation therapies on proprioceptive function. Front. Hum. Neurosci. 9:120. 10.3389/fnhum.2015.00120 25784872 PMC4345814

[B19] Krebs H. I. Volpe B. T. (2015). Robotics: a rehabilitation modality. Curr. Phys. Med. Rehabil. Rep. 3, 243–247. 10.1007/s40141-015-0101-6 26955506 PMC4778734

[B20] Kwakkel G. Kollen B. J. Krebs H. I. (2008). Effects of robot-assisted therapy on upper limb recovery after stroke: a systematic review. Neurorehabil. Neural Repair 22, 111–121. 10.1177/1545968307305457 17876068 PMC2730506

[B21] Latash M. L. (2012). The bliss of motor abundance. Exp. Brain Res. 217, 1–5. 10.1007/s00221-012-3000-4 22246105 PMC3532046

[B22] Lee D. D. Seung H. S. (2001). Algorithms for non-negative matrix factorization, in Advances in Neural Information Processing Systems 13, eds Leen T. K. Dietterich T. G. Tresp V. (Cambridge, MA: MIT), 556–562.

[B23] Lencioni T. Jonsdottir J. Cattaneo D. Crippa A. Gervasoni E. Rovaris M.. (2016). Are modular activations altered in lower limb muscles of persons with multiple sclerosis during walking? Evidence from muscle synergies and biomechanical analysis. Front. Hum. Neurosci. 10:620. 10.3389/fnhum.2016.00620 28018193 PMC5145858

[B24] Lo A. C. Guarino P. D. Richards L. G. Haselkorn J. K. Wittenberg G. F. Federman D. G.. (2010). Robot-assisted therapy for long-term upper-limb impairment after stroke. N. Engl. J. Med. 362, 1772–1783. 10.1056/NEJMx110075 20400552 PMC5592692

[B25] Lunardini F. Casellato C. Bertucco M. Sanger T. Pedrocchi A. (2017). Children with and without dystonia share common muscle synergies while performing writing tasks. Ann. Biomed. Eng. 45, 1949–1962. 10.1007/s10439-017-1838-0 28560552 PMC5531077

[B26] Maciejasz P. Eschweiler J. Gerlach-Hahn K. Jansen-Troy A. Leonhardt S. (2014). A survey on robotic devices for upper limb rehabilitation. J. Neuroeng. Rehabil. 11:3. 10.1186/1743-0003-11-3 24401110 PMC4029785

[B27] Mehrholz J. Hädrich A. Platz T. Kugler J. Pohl M. (2012). Electromechanical and robot-assisted arm training for improving generic activities of daily living, arm function, and arm muscle strength after stroke. Cochrane Database Syst. Rev. CD006876. 10.1002/14651858.CD006876.pub3 22696362

[B28] Mehrholz J. Pohl M. Platz T. Kugler J. Elsner B. (2015). Electromechanical and robot-assisted arm training for improving activities of daily living, arm function, and arm muscle strength after stroke. Cochrane Library. CD006876 10.1002/14651858.CD006876.pub4 PMC6465047 26559225

[B29] Overduin S. A. d'Avella A. Roh J. Carmena J. M. Bizzi E. (2015). Representation of muscle synergies in the primate brain. J. Neurosci. 35, 12615–12624. 10.1523/JNEUROSCI.4302-14.2015 26377453 PMC4571600

[B30] Pellegrino L. Coscia M. Muller M. Solaro C. Casadio M. (2018). Evaluating upper limb impairments in multiple sclerosis by exposure to different mechanical environments. Sci. Rep. 8:2110. 10.1038/s41598-018-20343-y 29391520 PMC5794735

[B31] Pirondini E. Coscia M. Marcheschi S. Roas G. Salsedo F. Frisoli A.. (2016). Evaluation of the effects of the Arm Light Exoskeleton on movement execution and muscle activities: a pilot study on healthy subjects. J. Neuroeng. Rehabil. 13:9. 10.1186/s12984-016-0117-x 26801620 PMC4724067

[B32] Pollock A. Farmer S. E. Brady M. C. Langhorne P. Mead G. E. Mehrholz J. (2014). Interventions for improving upper limb function after stroke *Cochrane Database* Syst. Rev. CD010820 10.1002/14651858.CD010820.pub2 PMC6469541 25387001

[B33] Posteraro F. Crea S. Mazzoleni S. Berteanu M. Ciobanu I. Vitiello N.. (2017). Technologically-advanced assessment of upper-limb spasticity: a pilot study. Eur. J. Phys. Rehabil. Med. 54, 536–544. 10.23736/S1973-9087.17.04815-8 28870058

[B34] Potter K. A. Fulk G. D. Salem Y. Sullivan J. E. Andrews A. W. Lanzino D. (2011). Outcome measures in neurological physical therapy practice. J. Neurol. Phys. Ther. 35, 57–64. 10.1097/NPT.0b013e318219a51a 21934360

[B35] Roh J. Rymer W. Z. Beer R. F. (2015). Evidence for altered upper extremity muscle synergies in chronic stroke survivors with mild and moderate impairment. Front. Hum. Neurosci. 9:6. 10.3389/fnhum.2015.00006 25717296 PMC4324145

[B36] Safavynia S. A. Torres-Oviedo G. Ting L. H. (2011). Muscle synergies: implications for clinical evaluation and rehabilitation of movement. Top. Spinal Cord Inj. Rehabil. 17, 16–24. 10.1310/sci1701-16 21796239 PMC3143193

[B37] Scano A. Chiavenna A. Caimmi M. Malosio M. Tosatti L. M. Molteni F. (2017). Effect of human-robot interaction on muscular synergies on healthy people and post-stroke chronic patients, in 15th IEEE International Conference on Rehabilitation Robotics (London: ICORR). 10.1109/ICORR.2017.8009302 28813874

[B38] Schmidt R. A. (1975). A schema theory of discrete motor skill learning. Psychol. Rev. 82:225 10.1037/h0076770

[B39] Tresch M. C. Cheung V. C. K. Avella A. Tresch M. C. Cheung V. C. K. Avella A. (2016). Matrix factorization algorithms for the identification of muscle synergies : evaluation on simulated and experimental data sets matrix factorization algorithms for the identification of muscle synergies : evaluation on simulated and experimental data sets. J. Neurophysiol. 95, 2199–2212. 10.1152/jn.00222.2005 16394079

[B40] Tropea P. Monaco V. Coscia M. Posteraro F. Micera S. (2013). Effects of early and intensive neuro-rehabilitative treatment on muscle synergies in acute post-stroke patients: a pilot study. J. Neuroeng. Rehabil. 10:103. 10.1186/1743-0003-10-103 24093623 PMC3850948

[B41] Winstein C. J. Stein J. Arena R. Bates B. Cherney L. R. Cramer S. C.. (2016). Guidelines for adult stroke rehabilitation and recovery: a guideline for healthcare professionals from the American Heart Association/American Stroke Association. Stroke 47, e98–e169. 10.1161/STR.0000000000000098 27145936

[B42] Wolpert D. M. Kawato M. (1998). Multiple paired forward and inverse models for motor control. Neural Netw. 11, 1317–1329. 10.1016/S0893-6080(98)00066-5 12662752

[B43] Wolpert D. M. Miall R. C. Kawato M. (1998). Internal models in the cerebellum. Trends Cogn. Sci. 2, 338–347. 10.1016/S1364-6613(98)01221-2 21227230

